# RIFTA: A Robust Iterative Fourier Transform-based dwell time Algorithm for ultra-precision ion beam figuring of synchrotron mirrors

**DOI:** 10.1038/s41598-020-64923-3

**Published:** 2020-05-18

**Authors:** Tianyi Wang, Lei Huang, Hyukmo Kang, Heejoo Choi, Dae Wook Kim, Kashmira Tayabaly, Mourad Idir

**Affiliations:** 1 National Synchrotron Light Source II (NSLS-II), Brookhaven National Laboratory, PO Box 5000, Upton, NY 11973 USA; 2 James C. Wyant College of Optical Sciences, the University of Arizona, 1630 E. University Blvd., P.O. Box 210094, Tucson, AZ 85721-0094 USA

**Keywords:** Optics and photonics, Applied optics, Other photonics

## Abstract

With the rapid evolution of synchrotron X-ray sources, the demand for high-precision X-ray mirrors has greatly increased. Single nanometer profile error is required to keep imaging capability at the diffraction limit. Ion Beam Figuring (IBF), as a highly deterministic surfacing technique, has been used for ultra-precision finishing of mirrors. One crucial step that guides the IBF process is dwell time calculation. A valid dwell time solution should be non-negative and duplicate the shape of the desired removal map. Another important aspect is to minimize the total dwell time. In this study, we propose a Robust Iterative Fourier Transform-based dwell time Algorithm (RIFTA) that automatically fulfills these requirements. First, the thresholded inverse filtering in Fourier transform-based deconvolution is stabilized and automated by optimizing the threshold value using the Nelder-Mead simplex algorithm. Second, a novel two-level iterative scheme is proposed to guarantee the minimized total dwell time with its non-negativity at each dwell point. Third, a bicubic resampling is employed to flexibly adapt the calculated dwell time map to any IBF process intervals. The performance of RIFTA is first studied with simulation, followed by a comparison with the other state-of-the-art dwell time algorithms. We then demonstrate with an experiment that, using the dwell time calculated by the RIFTA, the total dwell time is shortened by a factor of two and the RMS in a 5 × 50 mm clear aperture was reduced from 3.4 nm to 1.1 nm after one IBF run, which proves the effectiveness and the efficiency of the proposed algorithm.

## Introduction

As the third and fourth generation X-ray synchrotron sources is rapidly developing toward fully diffraction limited X-ray sources, the requirement of mirror specifications in terms of smoothness and shapes has drastically increased. Single nanometer profile error is usually required to avoid destruction of the incoming wave front and keep imaging capabilities at the diffraction limit^1,2^. Conventional mechanical polishing techniques, however, can hardly achieve the required high-level surface quality. Therefore, Computer Controlled Optical Surfacing (CCOS) methods have been studied and developed^3^. Ion Beam Figuring (IBF)^4^, as a highly deterministic CCOS technique, has been applied for the ultra-precision finishing of optical surfaces^5–10^. It removes materials from an optical surface at atomic level by physical sputtering. Compared with conventional polishing methods, IBF has the advantages of non-contact nature, no mechanical load force, minimal surface or subsurface damage, and low edge effects.

## Convolution polishing model

**Figure 1:**
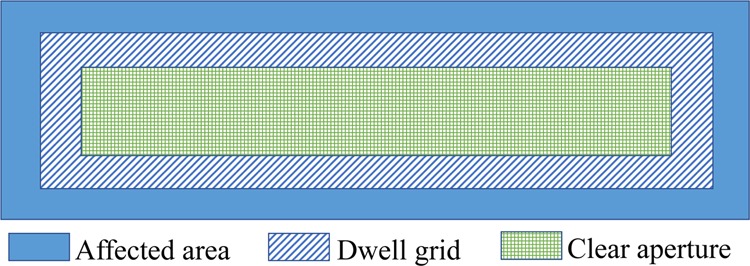
Schematic of the affected area, dwell grid, and clear aperture in an IBF process.

In the IBF (and any CCOS) process, as shown in Fig. 1, the Clear Aperture (CA) is specified as the useful area of a mirror surface that needs to be polished. Based on the convolution polishing model^3^, the CA should be usually enclosed in a larger Dwell Grid (DG) to resolve edge effects^10–14^. The removed height *z*(*x*, *y*) in DG, as described above, is therefore modeled as the convolution between the ion Beam Removal Function (BRF) *b*(*x*, *y*) and the dwell time map *t*(*x*, *y*) as

1 $$z(x,y)=b(x,y)\ast t(x,y),$$

where “*” denotes the convolution operation. To obtain a complete calculation result in the CA, the DG should be always larger than the outline perimeter of the CA with the radius of the BRF. While *b*(*x*, *y*) can be extracted from ion beam footprints bombarded on a mirror surface^10^ or via a Faraday cup, *z*(*x*, *y*) is calculated as the difference between the measured surface profile *z*_*m*_(*x*, *y*) and the desired surface profile *z*_*d*_(*x*, *y*) as *z*(*x*, *y*) = *z*_*m*_(*x*, *y*) − *z*_*d*_(*x*, *y*). Thus, the calculation of the dwell time map *t*(*x*, *y*) is a deconvolution process, which is an ill-posed inverse problem and may not have a unique solution^11,13,15^.

## Existing dwell time algorithms

Accurate calculation of *t*(*x*, *y*) is a crucial step that guides the dynamics between the ion beam and the mirror surface. A valid dwell time solution should possess three main characteristics. First, it should be non-negative, since most IBF systems do not have material adding capabilities. Second, the dwell time map *t*(*x*, *y*) should closely duplicate the desired removal map *z*_*d*_(*x*, *y*). Last, the total dwell time is expected to be minimized for an efficient figuring process. To fulfill these requirements, three categories of dwell time algorithms, namely the Fourier transform-based algorithm^14,16^, the matrix-based algorithms^10,11,13,17^, and the Bayesian-based algorithm^12^, have been attempted in IBF. The algorithms are briefly reviewed in the following paragraphs. Details are given in the “Methods” section.

Wilson and McNeil proposed the IBF dwell time algorithm based on the Fourier transform^14^ by transferring the deconvolution between *z*(*x*, *y*) and *b*(*x*, *y*) into point-wise division in frequency domain. Using Fast Fourier Transform (FFT), this algorithm is very computationally efficient. The non-negativity of *t*(*x*, *y*) is automatically guaranteed if *z*(*x*, *y*) is piston-adjusted to be non-negative. However, since the Fourier transform of *b*(*x*, *y*) is the denominator, the close-to-zero frequencies in its spectrum may have a noise amplification effect when performing the division. To solve this problem, a thresholded inverse filter is employed, where a threshold value *γ* is introduced to filter the small frequencies. One main disadvantage of this algorithm is that the dwell time calculation must be performed in the entire DG. The figuring results in CA will thus be highly affected when the profile error in DG is large. In this case, much more dwell time should be consumed to correct the profile error in DG so that the total dwell time will increase. In addition, to apply FFT, *z*_*d*_(*x*, *y*) and *t*(*x*, *y*) must have the same sampling interval^13^, however, a mismatch typically exists between practical metrology sampling and IBF’s motion control resolution.

Alternative dwell time algorithms that partially resolve these issues have been attempted. The matrix-based algorithms^10,11,13,17^ allow *z*_*d*_(*x*, *y*) and *t*(*x*, *y*) to have different sampling intervals by discretizing Eq. (1) in matrix form as

2 $$z({x}_{k},{y}_{k})=\mathop{\sum }\limits_{i=1}^{{n}_{t}}\,b({x}_{k}-{\xi }_{i},{y}_{k}-{\eta }_{i})t({\xi }_{i},{\eta }_{i}),$$

where *n*_*t*_ is the total number of dwell positions, *b*(*x*_*k*_ − *ξ*_*i*_, *y*_*k*_ − *η*_*i*_) is the material removal amount per unit time at (*x*_*k*_, *y*_*k*_) when the beam dwells at (*ξ*_*i*_, *η*_*i*_), and *t*(*ξ*_*i*_, *η*_*i*_) is the dwell time. This discretization brings the benefit that, instead of using the entire DG, only the CA information is needed. In other words, *z*(*x*_*k*_, *y*_*k*_) in Eq. (2) can be redefined as the height removed in the CA, *i.e. z*_*ca*_(*x*_*k*_, *y*_*k*_). In this sense, the dwell time solution will not be influenced by the shape outside the CA. Since the matrix is always ill-conditioned and rank-deficient, however, conventional Gaussian elimination cannot be applied. Singular Value Decomposition (SVD) has been used to find a least squares solution, but the small singular values also cause the noise amplification problem. Zhou *et al* thus proposed a Truncated SVD (TSVD) algorithm^17^, in which only the largest *k* singular values were kept. Nonetheless, both the computational and memory burdens of SVD are too heavy, restricting its wider applications in calculating large dwell time maps. Carnal *et al*. and Wu *et al*. employed a much more efficient LSQR algorithm^11,13^ to solve the linear system by introducing a damping factor *λ*. Although the computation speed has been largely increased, the piston adjustment has to be performed multiple times to guarantee the non-negativity of the dwell time map. Also, the calculated dwell time map *t*(*x*, *y*) can hardly duplicate the shape of *z*_*d*_(*x*, *y*)^10^. Recently, we proposed a Constrained Linear Least Squares (CLLS) algorithm for one dimensional IBF^10^. Instead of performing piston adjustment, the non-negativity was enforced by the lower-bound constraints. The inequality constraints were also added to control the local distribution of the dwell time, making sure *t*(*x*, *y*) smoothly duplicate the shape of *z*_*d*_(*x*, *y*). However, the computational burden became even heavier due to the introduction of the constraint matrices.

Jiao *et al*. viewed the deconvolution from the Bayesian perspective, in which a Poisson distribution of *b*(*x*, *y*)*t(*x*, *y*) and a uniform distribution of *z*(*x*, *y*) were assumed^12^. The Richardson-Lucy multiplicative algorithm^18^ was then applied to solve *t*(*x*, *y*) by iteratively maximizing a posteriori. A total variation norm weighted by a parameter *α* was also added to the optimization objective function to further improve the stability. This algorithm employed FFT to perform convolution, but it avoided the frequency-domain division in the Fourier transform-based algorithm. The non-negativity of *t*(*x*, *y*) was automatically guaranteed if the initial guess was non-negative. However, similar to the Fourier transform-based algorithm, the same sampling interval between *z*_*d*_(*x*, *y*) and *t*(*x*, *y*) was assumed.

It is also worth mentioning that, one common issue of the above algorithms is that each of them contains a *hyper-parameter*. The threshold value *γ* in the Fourier transform-based algorithm, the number of singular values *k* in TSVD, the damping coefficient *λ*, and the weight *α* for the total variation regularization in the Bayesian algorithm are all required to be preset. These hyper-parameters are always hard (and subjective) to set, which affects the robustness of the algorithms.

## RIFTA

In this study, we propose a novel Robust Iterative Fourier Transform-based dwell time Algorithm (RIFTA) that can be applied to IBF (and any CCOS techniques requiring dwell time optimization). It takes the advantages of both the Fourier transform-based and the matrix-based algorithms while mitigates their problems. First, the Nelder-Mead simplex algorithm^19^ is employed to automatically optimize *γ* for the inverse filtering process in the Fourier-based algorithm. Therefore, no hyper-parameter is needed in the RIFTA, which is convenient to use and robust in performance.

Furthermore, a two-level iterative scheme guarantees the non-negativity of the dwell time with the minimal penalty, *i.e*. increase, in the total dwell time. The inner-level iterations only utilize the shape error in the CA to calculate the dwell time. Therefore, the final dwell time map is not influenced by the shape error outside the CA and the total dwell time is decreased compared with the one calculated on the entire DG. However, different from the matrix-based algorithms, the dwell time map calculated using only the CA information is not accurate enough so that the inner-level iterations keep updating the dwell time map according to the estimated residual in the CA until the specified accuracy level is achieved. The outer-level iterations are employed to further reduce the total dwell time by minimizing the size of the DG. This is also helpful for the other CCOS techniques using physical polishing tools that may overhang at the edges.

Last, bicubic resampling is introduced to flexibly adapt the calculated dwell time to any practical sampling intervals. In the following section, the performance of the three strategies used in RIFTA is first studied step-by-step on simulated surface error maps. The calculation results using the existing dwell time algorithms mentioned above are then given as a comparison, showing the superiority of RIFTA over these algorithms. Finally, a proof-of-concept IBF experiment on a 5 × 50 mm CA is demonstrated. Using the RIFTA, the total dwell time has been reduced by a factor of two compared the one calculated using the Fourier transform-based method. The RMS in the CA has been reduced from 3.4 nm to 1.1 nm after one IBF run, which proves the effectiveness and efficiency of the RIFTA.

## Results

The effectiveness of the proposed RIFTA is first verified using simulation, followed by an IBF experiment. To separate the experimental results from the simulation, two different colormaps are used in the simulation and experiment figures, respectively.

### Simulation

**Figure 2:**
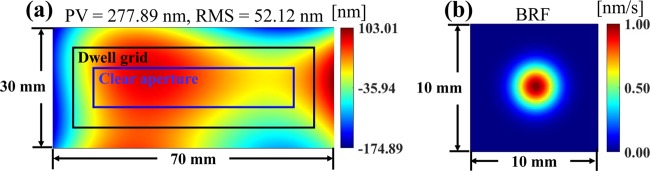
Simulated surface error map (**a**) and Gaussian BRF (**b**).

As shown in Fig. 2(a), a 30 × 70 mm rectangular surface error map with 277.89 nm Peak-to-Valley (PV) and 52.12 nm RMS is generated using the Legendre polynomials^20^ with the coefficients *Q*_4_ = −1, *Q*_6_ = −1, *Q*_7_ = 2, *Q*_9_ = −1, and *Q*_10_ = −0.5. The sampling interval is 0.12 mm and a Gaussian white noise with 0.3 nm Standard Deviation (STD) is added. The Gaussian BRF, as shown in Fig. 2(b), has a peak removal rate of 1 nm/s and a radius *r*_*b*_ = 5 mm.

#### Result after *gamma* optimization

**Figure 3:**
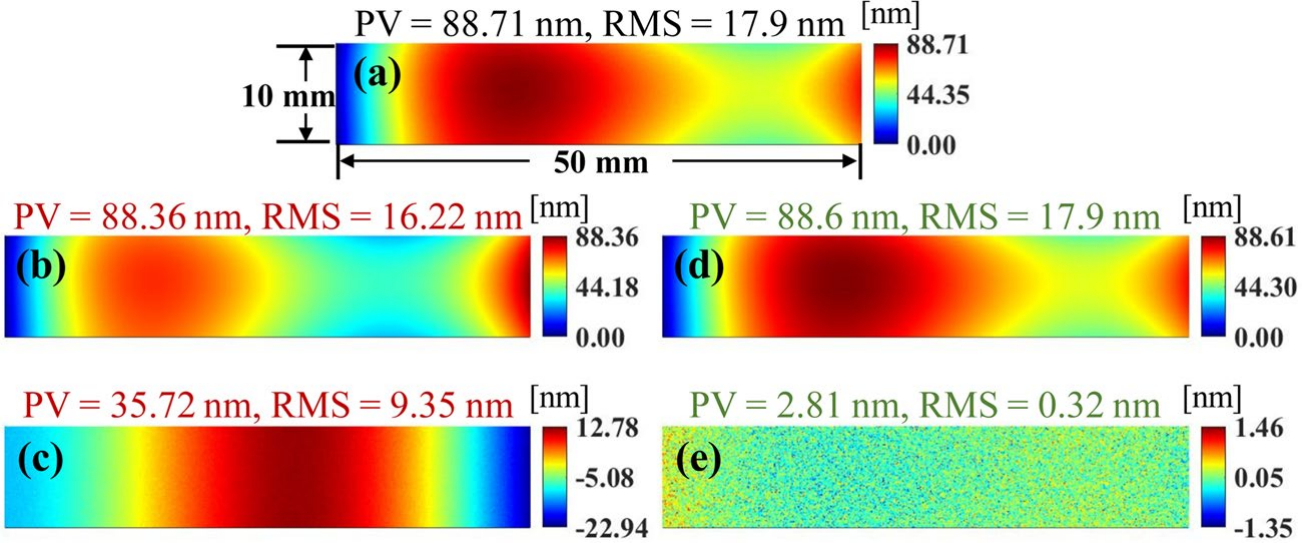
Calculation results in CA using *γ*_*ini*_ and *γ*_*opt*_: (**a**) desired height removal map; (**b**) removed height using *γ*_*ini*_; (**d**) residual using *γ*_*ini*_; (**c**) removed height using *γ*_*opt*_; and (**e**) residual using *γ*_*opt*_.

The results of *γ* optimization is demonstrated in Fig. 3. The size of the CA is set as 10 × 50 mm (see Fig. 3(a)). The size of the DG is fixed at 20 × 60 mm. The two-level iterative scheme is temporarily disabled to demonstrate the *γ* optimization process only. Figure 3(b,c) show the removed height and the residual in the CA calculated using *γ*_*ini*_, which is the initial guess calculated by the method explained in the “Methods” section. The PV and RMS of the residual in the CA remain at 35.72 nm and 9.35 nm, respectively, which indicates that a large portion of material is not removed. After *γ* optimization based on the Nelder-Mead algorithm, the calculation results using *γ*_*opt*_ are shown in Fig. 3(d,e). The PV and RMS of the residual in the CA reduces to 2.81 nm and 0.32 nm, respectively, showing a significant improvement. It is also important to note that the 0.32 nm RMS coincides with the preset noise STD (0.3 nm), which gives the theoretical limit of the optimization in our simulation.

#### Performance of the two-level iterative scheme

**Figure 4:**
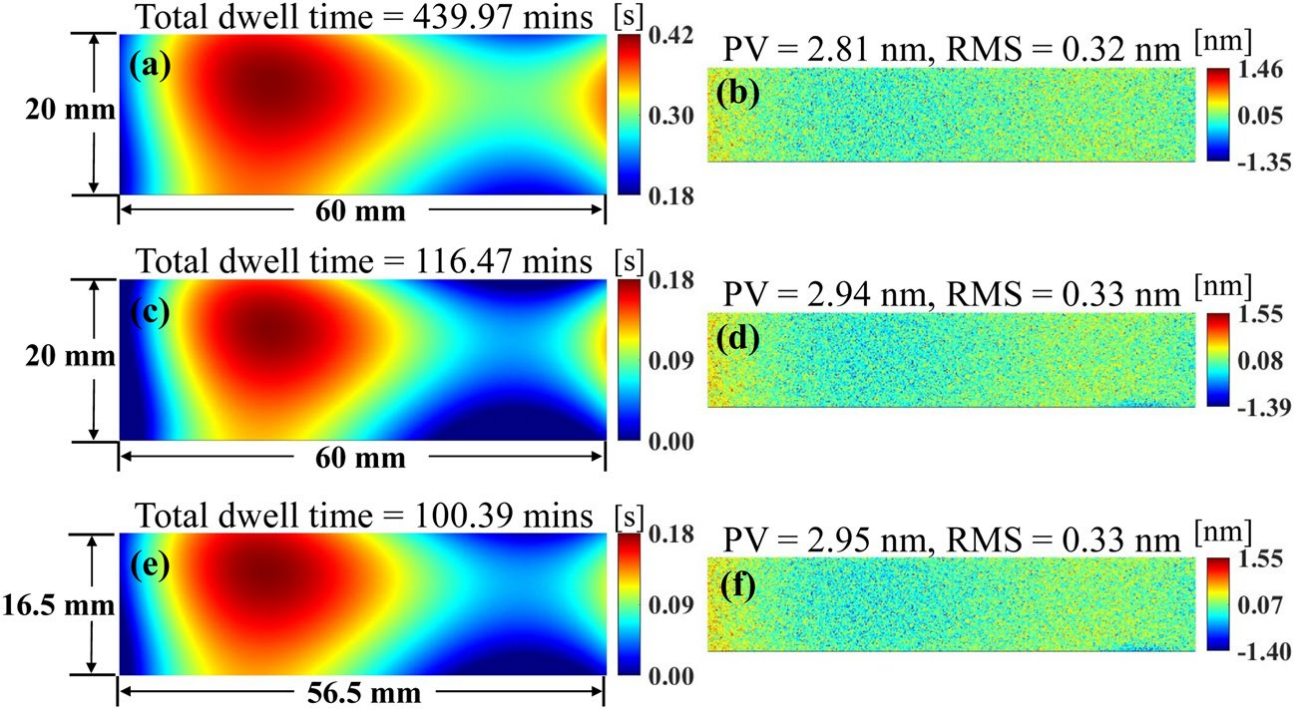
Calculated dwell time maps (**a**,**c**,**e**) and residual maps in CA (**b**,**d**,**f**) without the two-level iterative scheme (**a**,**b**), with only inner iterations (**c**,**d**), and with the two-level iterative scheme (**e**,**f**).

The effectiveness and efficiency of the two-level iterative scheme is further studied in Fig. 4. As shown in Fig. 4(a), the total dwell time is 439.97 mins without using the two-level iterative scheme. As shown in Fig. 4(b,d), after applying the inner iterations, the total dwell time is greatly reduced to 116.47 mins (about 74% shorter) without affecting the obtained performances in the CA. It is also worth mentioning that the inner iterations only costs 11.9 s (using a computer with an Intel Xeon Gold 5118 CPU and 64 GB RAM) so that they can be efficiently included to the outer iterations for the DG minimization. As shown in Fig. 4(e), the total dwell time can be further reduced to 100.39 mins with a smaller DG size of 56.5 × 16.5 mm after the outer iterations are employed, while the residual increment in the CA is negligible.

#### Downsampling the dwell time map to 1 mm sampling interval

**Figure 5:**
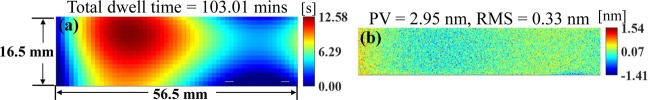
Dwell time map resampled to 1 mm sampling interval (**a**) and the corresponding residual map in CA (**b**).

The dwell time maps calculated in Fig. 4(a,c,e) have the same sampling interval (0.12 mm) as the simulated surface error map in Fig. 2(a). In this case, the dwell time at each dwell point is less than 0.5 s, which demands extremely low-latency and high-acceleration IBF motion hardware. To implement the calculated dwell time map in Fig. 4(e), it is re-sampled to 1 mm sampling interval (see Fig. 5(a)), in which the second-order dwell time at each dwell point is more practical to execute. The resultant residual map in the CA is shown in Fig. 5(b), which indicates that the algorithmic accuracy is not affected by the re-sampling operation compared with Fig. 4(f).

#### Comparison between RIFTA and the existing dwell time algorithms

**Figure 6:**
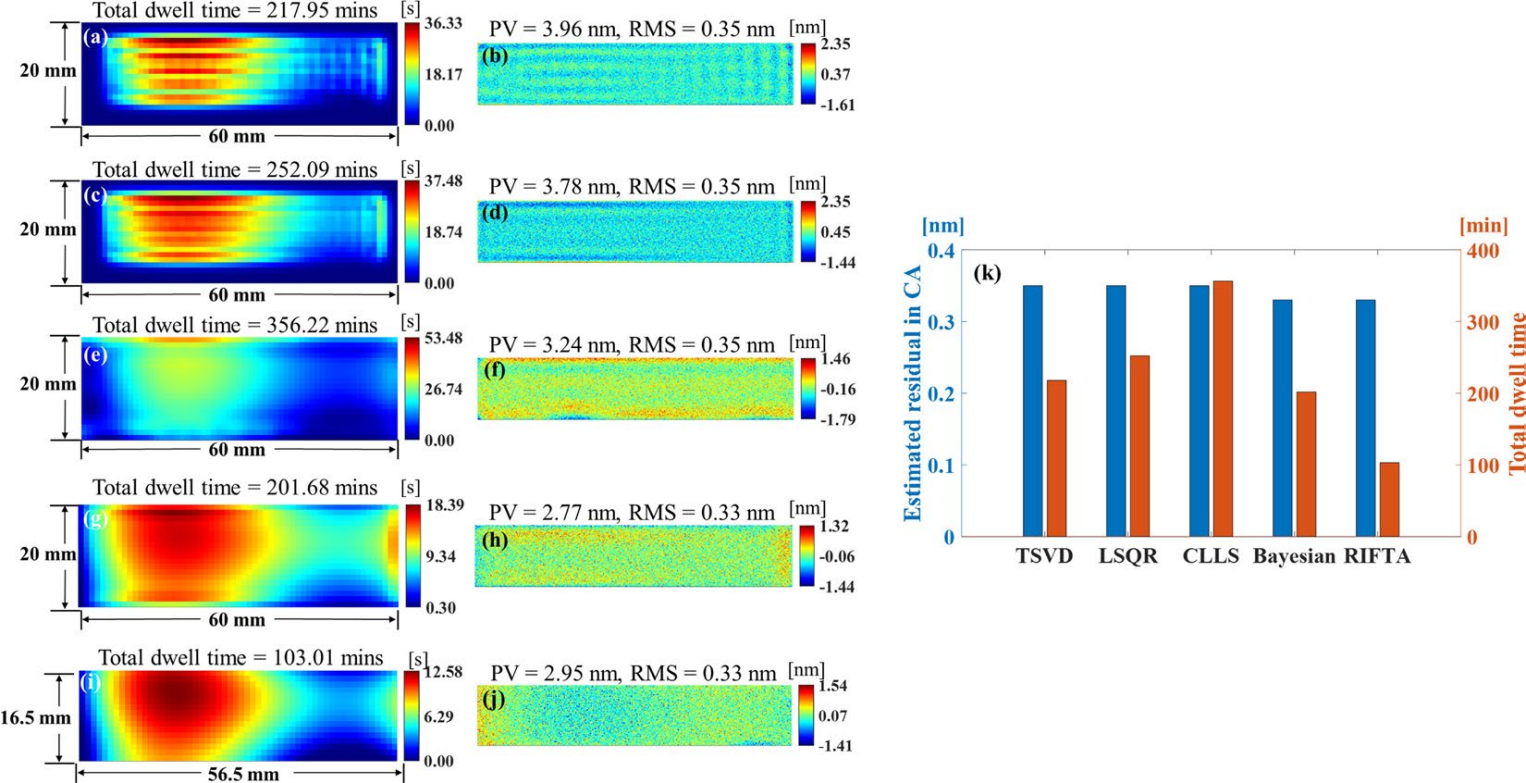
Dwell time maps and estimated residual maps in the CA calculated using TSVD (**a**,**b**), LSQR (**c**,**d**), CLLS (**e**,**f**), Bayesian-based algorithm (**g**,**h**), and RIFTA **(i**,**j**). The RIFTA achieves the best performance in terms of both the smallest estimated residual in the CA and the shortest total dwell time (**k**).

The dwell time maps calculated using TSVD^17^, LSQR^11,13^, CLLS^10^, and the Bayesian-based algorithm^12^ are shown in Fig. 6(a,c,e,g). The corresponding estimated residual map in the CA are given in Fig. 6(b,d,f,h). The same 1 mm processing interval is used in all the different algorithms. The same re-sampling strategy described in the “Results” section is applied in the Bayesian-based algorithm.

It can be found that the RMS of the estimated residuals in the CA calculated by all the four algorithms coincide with the preset noise level (*i.e* 0.3 nm). However, it is obvious that the the dwell time maps calculated by TSVD and LSQR hardly duplicate the shape of *z*_*d*_(*x*, *y*) in the DG. With the constraints on local distributions of the dwell time, the shape of the dwell time map calculated by CLLS algorithm is closer to the shape of *z*_*d*_(*x*, *y*). However, this benefit results in a much longer total dwell time than the other three. The dwell time map calculated by the Bayesian-based algorithm is the best among the four in terms of both the shortest total dwell time and the closest duplication of the shape of *z*_*d*_(*x*, *y*). Nonetheless, it still cannot match the RIFTA’s performance shown in Fig. 6(i,j). The final estimated residual in the CA and the total dwell time calculated by the algorithms are shown in Fig. 6(k). It is obvious that the RIFTA achieves the smallest estimated residual in the CA with the shortest total dwell time.

### Experiment

**Figure 7:**
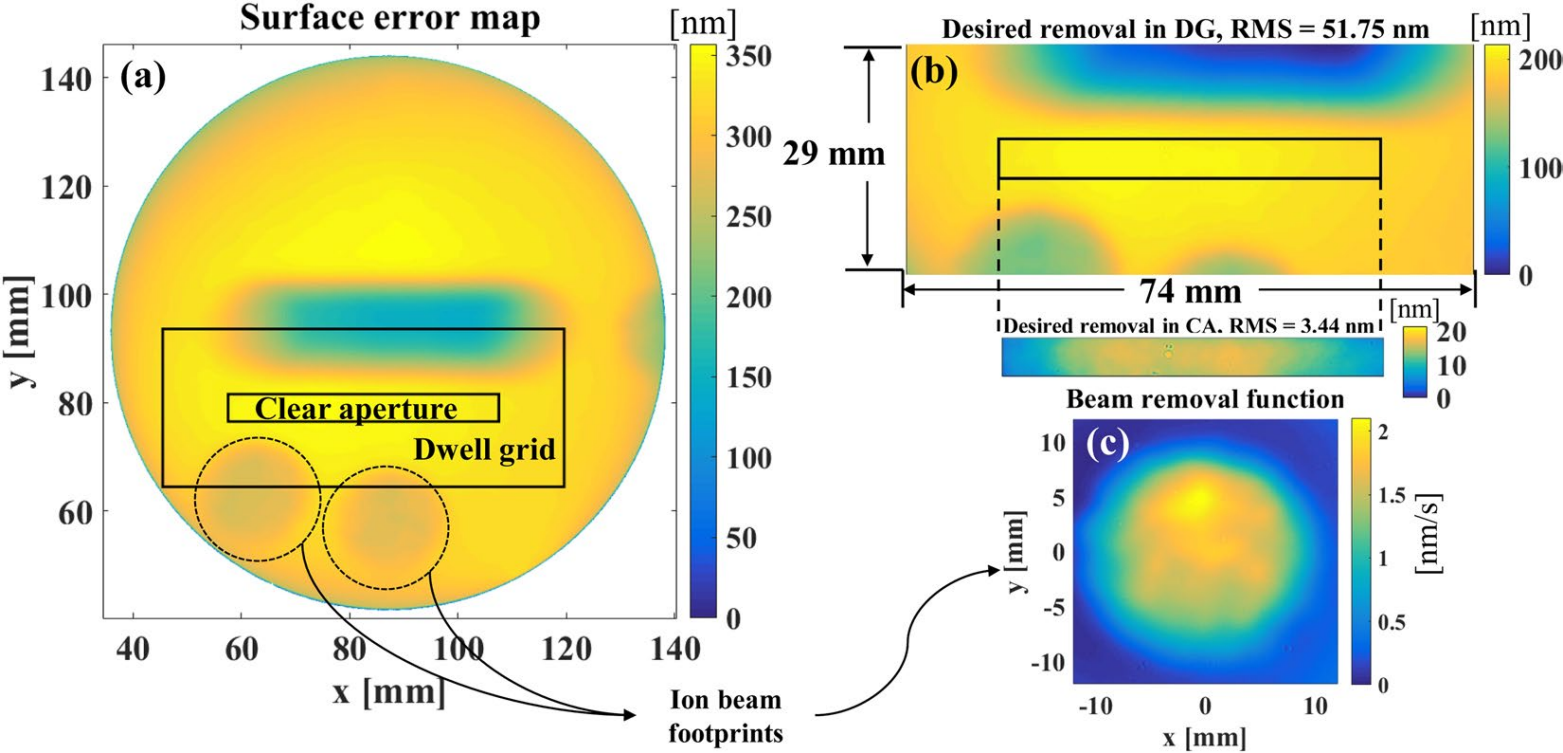
(**a**) Surface error map of a silicon circular flat mirror; (**b**) the error maps in the dwell grid (DG) and the clear aperture (CA); and (**c**) the beam removal function obtained from the bombarded ion beam footprints.

As a proof-of-concept experiment, the proposed RIFTA has been applied to a real IBF process. We used a circular flat Silicon mirror with a 5 × 50 mm CA as shown in Fig. 7(a). This mirror was measured with a Zygo Verifire interferometer with a 0.13 mm/pixel lateral resolution. This mirror was initially used to study the parameters of the ion source and test its stability so that some patterns already exist on the mirror surface. The ion source used in our IBF system is a Kaufman & Robinson KDC10 coupled with a LFN1000 neutralizer. The IBF process parameters are beam voltage, *V*_*b*_ = 600 V; beam current, *I*_*b*_ = 10 mA; accelerator voltage, *V*_*a*_ = −90 V; accelerator current, *I*_*a*_ = 2 mA; and LFN emission current, *I*_*e*_ = 10 mA. Two ion beam footprints bombarded at the bottom were used to estimate the BRF (see Fig. 7(c)) and the trench scanned in the center along the *x* axis was for studying the stability of the ion beam.

As mentioned in the “Introduction” section, one advantage of the inner-level iterations of RIFTA is that the final dwell time solution will not be affected by the large shape errors outside the CA. To verify this, the CA in this experiment is defined as a center 5 × 50 mm region between the trench and the two footprints (see Fig. 7(a)). The extracted DG and CA error maps are given in Fig. 7(b). It can be found that the shape error in the DG is 51.75 nm RMS, which is much larger than the 3.44 nm in the CA. The BRF used in this experiment, as shown in Fig. 7(c), is extracted from the bombarded footprints on the mirror in Fig. 7(a). The diameter of the BRF is 24 mm so that the initial size of the DG is 29 × 74 mm.

**Figure 8:**
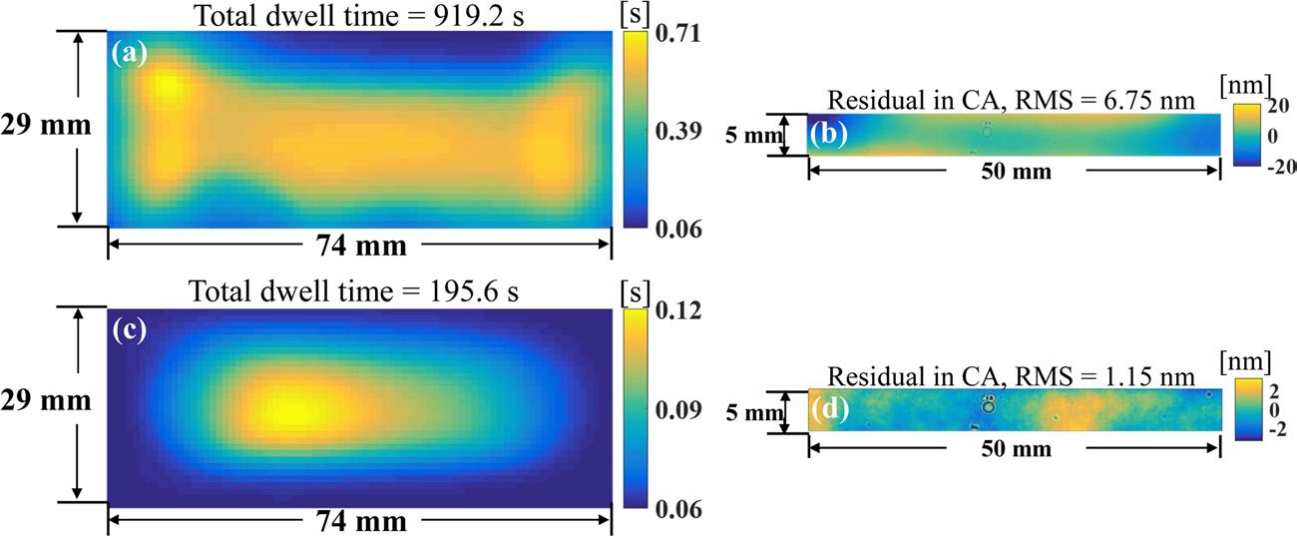
Dwell time maps (**a**,**c**) and estimated residual maps in the CA (**b**,**d**) calculated using the Bayesian-based algorithm (**a**,**b**) and the TSVD algorithm (**c**,**d**).

The Bayesian-based algorithm and the TSVD algorithm are first applied to calculate the dwell time solutions for the CA defined in Fig. 7(b). The calculated dwell time maps are shown in Fig. 8(a,c). The corresponding estimated residual in the CA are given in Fig. 8(b,d). It can be observed that the total dwell time calculated by the Bayesian-based algorithm is very high due to the influence of the large shape error in the DG. This also leads to a bad estimated residual map (*i.e*. 6.75 nm RMS) in the CA. The TSVD, on the other hand, only utilizes the CA’s information so that the total dwell time required is shorter and the estimated residual in the CA has been reduced from 3.44 nm to 1.15 nm RMS. It is worth noting that, however, both the dwell time maps do not duplicate the desired removal map in Fig. 7(b).

**Figure 9:**
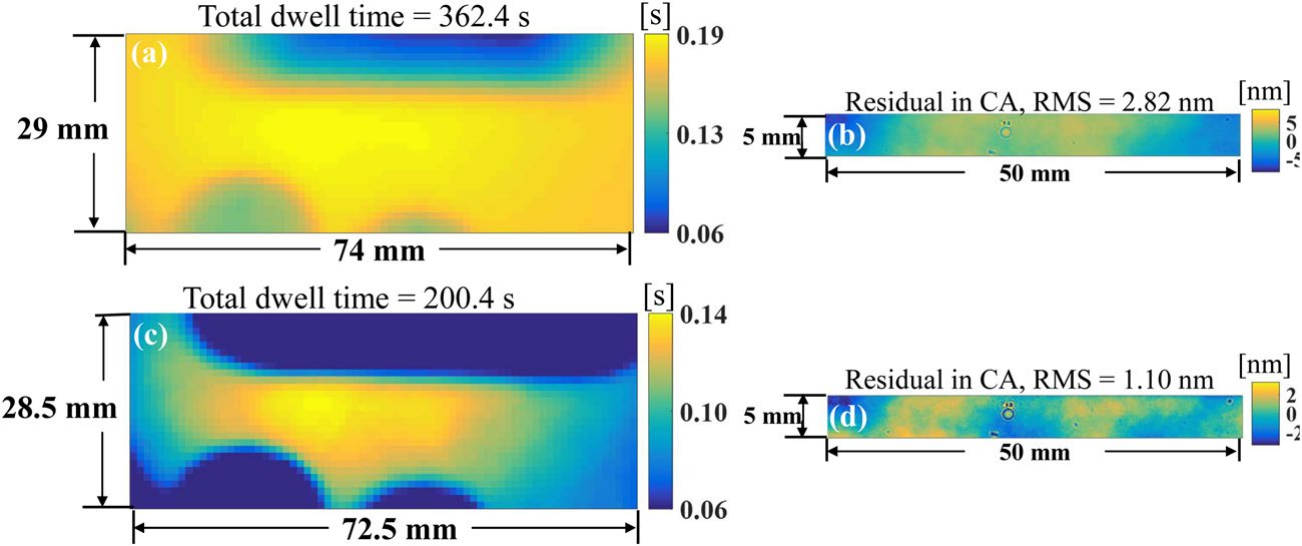
Dwell time maps (**a**,**c**) and estimated residual maps in the CA (**b**,**d**) calculated using the RIFTA without the two-level iterative scheme (**a**,**b**) and with the two-level iterative scheme (**c**,**d**).

The proposed RIFTA then is used to calculate the dwell time solution. Initially, the dwell time solution is obtained by the RIFTA without the two-level iterative scheme. The resultant dwell time map is shown in Fig. 9(a) and the corresponding estimated residual map in the CA is given in Fig. 9(b). The dwell time map smoothly duplicate the shape of the desired removal in the DG shown in Fig. 7(b), however, the 2.82 nm RMS estimated residual in the CA is not optimal. The two-level iterative scheme is then enabled to optimize the dwell time solution only based on the CA while minimize the size of the DG. It can be found in Fig. 9(c) that the large shape error in the DG has been avoided and the size of the DG has been shrunk to 28.5 × 72.5 mm. Also, the total dwell time has been reduced from 362.4 s to 200.4 s. The estimated residual in the CA, as shown in Fig. 9(d), has been reduced to 1.10 nm, which is the lowest among all the presented results. Therefore, the dwell time solution demonstrated in Fig. 9(c) has been applied to the real IBF process below.

**Figure 10:**
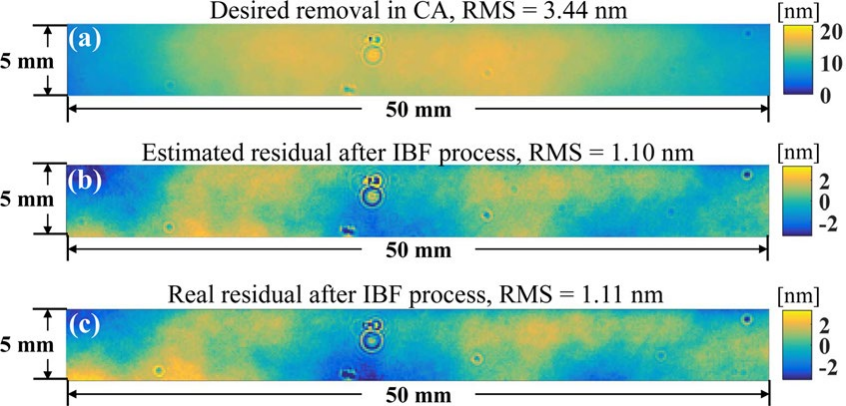
(**a**) Desired removal map in the CA; (**b**) estimated residual map in the CA; and (**c**) real residual map in the CA after one IBF run.

Using the dwell time map calculated by the RIFTA (see Fig. 9(c)), one IBF run has been performed on the CA shown in Fig. 10(a). The real residual shape error obtained is 1.11 nm shown in Fig. 10(c), as expected in our simulation estimation shown in Fig. 10(b). It is also worth mentioning that, the shapes of the estimation and the real residual map in the CA are very similar to each other, which proves the effectiveness of the real application of the proposed RIFTA. The mirror roughness before and after the IBF process is measured using a Zygo New View white-light microscope interferometer. Five points of interest located in the CA of are measured. The RMS roughness before and after the IBF process are 0.30 nm, 0.31 nm, 0.34 nm, 0.30 nm, and 0.33 nm versus 0.31 nm, 0.32 nm, 0.32 nm, 0.32 nm, and 0.33 nm, which demonstrate that the surface roughness of the mirror is almost not affected by the IBF process.

## Discussion

A good dwell time algorithm for Ion Beam Figuring (IBF) (and other Computer Controlled Optical Surface (CCOS) techniques) should contain the following crucial characteristics. First and foremost, the calculated dwell time should be non-negative, since IBF (and most CCOS techniques) often does not have the material adding capability. Moreover, the calculated dwell time should be able to be robustly applied to obtain expected figuring results. One key criterion of evaluating the robustness is by examining if the calculated dwell time map smoothly duplicates the desired removal map while the estimated residual error in the CA is small. Additionally, the total dwell time should be minimized to guarantee the stability of the ion source and achieve high-efficiency figuring process. Last but not least, the calculated dwell time map should be able to be flexibly adapted to the practical IBF process machine’s motion control resolution.

In order to fulfill these requirements, a new dwell time algorithm, RIFTA, is proposed in this study. First, a *γ* optimization method based on the Nelder-Mead simplex algorithm is proposed to stabilize the Fourier transform-based deconvolution and automate the *γ* tuning in the thresholded inverse filtering process. Furthermore, a novel two-level iterative scheme is employed to reduce the influence caused by the shape error outside the CA and minimize the total dwell time. The inner iterations ensure the non-negativity of the calculated dwell time while significantly reduce the total dwell time by iteratively updating the dwell time solution only based on the CA’s information. The outer iterations, can further shorten the total dwell time by minimizing the size of the DG. Last, the calculated dwell time is flexibly adapted to a practical sampling interval with bicubic resampling.

The performance of the RIFTA has been first studied by simulation. The comparison between the RIFTA and the other known dwell time algorithms demonstrates its superiority in the above- mentioned important aspects. The experimental results further demonstrate that the proposed RIFTA is robust, efficient, and effective dwell time algorithm that can be applied to real IBF applications.

It is also worth noting that, besides IBF, the RIFTA is generally applicable to any other CCOS techniques. However, one limitation of the current RIFTA is that the outer-level iterations cannot be applied to optimize the size of the DG if the CA is almost the entire mirror surface. Additionally, the two-level iterative scheme is more effective in reducing the total dwell time if the difference between the shape errors inside and outside the CA is larger. When the difference is very small, however, the reduction of the total dwell time may not be obvious. The MATLAB code for all the dwell time algorithms described in this paper has been uploaded to a public GitHub repository^21^.

## Methods

### Existing dwell time calculation methods

#### Fourier transform-based method

Wilson and McNeil proposed the pioneering IBF dwell time algorithm based on the Fourier transform^14^, in which *t*(*x*, *y*) is calculated as

3 $$t(x,y)={ {\mathcal F} }^{-1}\,\left[\frac{{Z}_{d}(u,v)}{B(u,v)}\right]$$

where *Z*_*d*_(*u*, *v*) and *B*(*u*, *v*) are the Fourier transforms of the desired removal *z*_*d*_(*x*, *y*) and *b*(*x*, *y*), respectively; ${ {\mathcal F} }^{-1}(\cdot )$ represents the inverse Fourier transform. However, as pointed out by Wu *et al*.^13^ and Jiao *et al*.^12^, Eq. (3) is unstable. The amplitudes of *B*(*u*, *v*) at certain frequencies can be very close to zero so that any noises in *Z*_*d*_(*u*, *v*) at those frequencies are enormously amplified, resulting in algorithm failure. Therefore, a thresholded inverse filter $\bar{B}(u,v;\gamma )$^14,22^ has been employed to substitute *B*(*u*, *v*) in Eq. (3) as

4 $$t(x,y;\gamma )={ {\mathcal F} }^{-1}\,\left[\frac{{Z}_{d}(u,v)}{\bar{B}(u,v;\gamma )}\right],$$

where

5 $$\bar{B}(u,v;\gamma )=\{\begin{array}{cc}B(u,v), & {\rm{if}}\,|B(u,v)| > \gamma \\ \gamma , & {\rm{otherwise}}\end{array},$$

and *γ* is the thresholded amplitude of *B*(*u*, *v*). In Eq. (5), *γ* serves as a threshold to make an “almost” full inverse instead of a full inverse of *B*(*u*, *v*). The *γ* values depend on what units are used in the calculation. Nonetheless, the determination of the optimal *γ* value is nontrivial and subjective.

#### TSVD algorithm

Equation (2) can be re-written in matrix form as

6 $$\begin{array}{cccc}\mathop{\underbrace{(\begin{array}{c}{z}_{0}\\ {z}_{1}\\ \vdots \\ {z}_{{N}_{r}-1}\end{array})}}\limits_{z} & = & \mathop{\underbrace{(\begin{array}{cccc}{b}_{1,1} & {b}_{1,2} & \ldots  & {b}_{1,{N}_{t}-1}\\ {b}_{1,1} & {b}_{2,2} & \ldots  & {b}_{2,{N}_{t}-1}\\ \vdots  & \vdots  & \vdots  & \vdots \\ {b}_{{N}_{r}-1,1} & {b}_{{N}_{r}-1,2} & \ldots  & {b}_{{N}_{r}-1,{N}_{t}-1}\end{array})}}\limits_{{\bf{B}}} & \mathop{\underbrace{(\begin{array}{c}{t}_{0}\\ {t}_{0}\\ \vdots \\ {t}_{{N}_{t}-1}\end{array})}}\limits_{t}\end{array}.$$

SVD can be used to obtain a minimum norm solution to Eq. (6) as

7 $${t}_{svd}=\mathop{\sum }\limits_{i=1}^{{N}_{r}}\,\frac{{{\bf{u}}}_{i}^{{\rm T}}{{\bf{z}}}_{d}}{{\sigma }_{i}}{{\bf{v}}}_{i},$$

where *σ*_*i*_, *i* = 1, 2, 3, …, *N*_*r*_ are the singular values appearing in non-increasing order; **u**_***i***_ and **v**_***i***_ are the left and right singular vectors of **B**, respectively. Note that, similar to the Fourier-based algorithm, the division of the small singular values in Eq. (7) is problematic^17^. Zhou *et al*. thus proposed to truncate the division of the small singular as

8 $${t}_{tsvd}=\mathop{\sum }\limits_{i=1}^{k}\,\frac{{{\bf{u}}}_{i}^{{\rm T}}{{\bf{z}}}_{d}}{{\sigma }_{i}}{{\bf{v}}}_{i},\,k\le {N}_{r}.$$

#### LSQR algorithm

Instead of performing the time-consuming SVD of the matrix **B**, damping has been attempted by applying the LSQR algorithm^11,13^, in which case the solution is

9 $${t}_{lsqr}=\mathop{\sum }\limits_{i=1}^{{N}_{r}}\,\frac{{\sigma }_{i}{{\bf{u}}}_{i}^{{\rm T}}{{\bf{z}}}_{d}}{{\sigma }_{i}^{2}+{\lambda }^{2}}{{\bf{v}}}_{i},$$

where *λ* is the damping parameter. LSQR is very computationally efficient and consumes much less memory space than SVD.

#### CLLS algorithm

It is worth noting that, the main problem of both the TSVD and LSQR algorithms is the piston adjustment of *z*(*x*, *y*) as *z*(*x*, *y*) + *Ψ* to guarantee the non-negativity of the calculated *t*(*x*, *y*), where *Ψ* is a constant piston value. Wang *et al*. proposed the CLLS algorithm, which does not require piston adjustment by modeling the deconvolution as

10 $$\begin{array}{ll}{\rm{minimize}} & \frac{1}{2}{||{\bf{B}}t-z||}_{2}^{2}\\ {\rm{subject}}\,{\rm{to}} & {\bf{A}}t\le b\\  & t\ge {{\bf{0}}}^{{\rm T}}\end{array}$$

where

11 $${\bf{A}}=(\begin{array}{llllll}1 & -1 &  &  &  & 0\\  & 1 & -1 &  &  & \\  &  & \ddots  & \ddots  &  & \\  &  &  & \ddots  & \ddots  & \\ 0 &  &  &  & 1 & -1\\ -1 & 1 &  &  &  & 0\\  & -1 & 1 &  &  & \\  &  & \ddots  & \ddots  &  & \\  &  &  & \ddots  & \ddots  & \\ 0 &  &  &  & -1 & 1\end{array}),$$

12 $$b={({b}_{0},{b}_{1},\mathrm{..}.,{b}_{{N}_{r}-2},{b}_{0},\mathrm{..}.,{b}_{{N}_{r}-2})}^{{\rm T}}$$

where *b*_*i*_ is the maximum absolute dwell time difference between each two consecutive machining positions *i* and *i* + 1 for *i* = 0, 1, 2, …, *N*_*r*_ − 2. The non-negativity is enforced by the lower-bound constraints while the inequalities constrained the local distribution of the dwell time. In the real experiments, however, the direct application of Eq. (10) results in unexpected results if the inequality constraints are too strict. Therefore, a coarse-to-fine scheme was employed. On the coarse level, looser constraints are applied to obtain a coarse result *t*_*coarse*_(*x*, *y*), which is then polynomial fitted as *t*_*fit*_(*x*, *y*). On the finer level, the required constraints are applied to the residual map calculated from obtained from the fitted map as ${z}_{r\_ca}={z}_{d}[{\rm{size}}(CA)]-{\bf{B}}\ast {t}_{fit}$. The final dwell time map is thus the addition of the coarse and the fine level results as *t*_*clls*_ = *t*_*coarse*_ + *t*_*fine*_.

#### Bayesian-based algorithm

Jiao *et al*. proposed the Bayesian-based dwell time algorithm for IBF^12^. Assume that *z*(*x*, *y*) and *t*(*x*, *y*) are both random, according to Bayesian theory, the relation among the posterior *P*(*t*|*z*), the prior *P*(*T*), and the likelihood *P*(*z*|*t*) *z*, *b* is

13 $$P(t|z)=P(z|t)\frac{P(t)}{P(z)}$$

Assume that *t*(*x*, *y*) follows the uniform distribution and *P*(*z*|*t*) is the Poisson distribution^18^, the dwell time can be solved by MAP as

14 $${{\rm{\min }}}_{t}{J}_{1}(t)$$

where

15 $${J}_{1}(t)=\int {\int }_{\Omega }\,(b\ast t-z\times \,\log (b\ast t))dxdy.$$

Setting ∇*J*_1_(*t*) = 0, Eq. (14) can be solved by a multiplicative algorithm^18^ as

16 $${t}_{k+1}={t}_{k}\times \left(\frac{z(-x,-y)}{\int {\int }_{\Omega }\,zdxdy}\ast \frac{z}{b\ast {t}_{k}}\right)$$

With Eq. (16), the non-negativity of the dwell time map is automatically guaranteed if the initial guess *t*_0_ is non-negative. To achieve faster convergence, a total variation regularization term ${J}_{2}(t)=\lambda \int {\int }_{\Omega }\,|\nabla t|dxdy$ is added so that the MAP problem was rewritten as

17 $${{\rm{\min }}}_{t}({J}_{1}+{J}_{2}),$$

which can be solved as

18 $${t}_{k+1}=\frac{{t}_{k}}{1-\lambda \frac{div(\nabla {t}_{k})}{|\nabla {t}_{k}|}}\times \left(\frac{z(-x,-y)}{\int {\int }_{\Omega }\,zdxdy}\ast \frac{z}{b\ast {t}_{k}}\right).$$

### RIFTA

#### Find the optimal *gamma*

Like the Fourier transform-based algorithm, the first step of RIFTA is to determine *γ* in Eqs. (4) and (5). Instead of setting an appropriate value for *γ*, it is found by an optimization process. We define a residual map *z*_*r*_(*x*, *y*; *γ*) to be the difference between the desired removal *z*_*d*_(*x*, *y*) and the removed height $z(x,y;\gamma )=b(x,y)\ast t(x,y;\gamma )$ as

19 $${z}_{r}(x,y;\gamma )={z}_{d}(x,y)-z(x,y;\gamma ).$$

The effectiveness of dwell time calculation can then be quantitatively evaluated by interrogating the RMS of *z*_*r*_ as *RMS*[*z*_*r*_]. Ideally, it should only reflect the measurement noise. Therefore, finding the optimal *γ*, *i.e. γ*_*opt*_, can be defined as an unconstrained optimization problem as

20 $${\gamma }_{opt}=\mathop{{\rm{argmin}}}\limits_{\gamma }\,RMS[{z}_{r}(x,y;\gamma )]\mathrm{}.$$

Substituting Eqs. (1), (4), and (19) to Eq. (20), the optimization can be reformulated as

21 $${\gamma }_{opt}=\mathop{{\rm{argmin}}}\limits_{\gamma }\,RMS\left\{{z}_{d}(x,y)-b(x,y)\ast { {\mathcal F} }^{-1}\left[\frac{{Z}_{d}(u,v)}{\bar{B}(u,v;\gamma )}\right]\right\}.$$

It can be observed that the optimization space in Eq. (21) is not smooth due to the thresholding operations in $\bar{B}(u,v;\gamma )$. Its gradients cannot be calculated so that any derivative-based optimization algorithms can hardly be applied. In RIFTA, as shown in Line 8 in Algorithm 4.2.2, we apply the Nelder-Mead algorithm^19^. It directly search for the optimal variables that minimize a scalar-valued non-linear function using only function values without any derivative information^23^. It is especially efficient when the number of variables is small^23^ and the computational complexity of an objective function is low^24^. Equation (21) is a scalar-valued non-linear function and *γ* is the only variable to be optimized. Also, the computational burden of Eq. (21) is not heavy thanks to the FFT algorithm. Therefore, the Nelder-Mead algorithm is an appropriate solver for *γ*_*opt*_. It is worth noting that, however, the Nelder-Mead algorithm requires a good initial guess to obtain a reasonable solution and a fast convergence rate. In our study, the initial value *γ*_*ini*_ is obtained as the ratio between *RMS*[*z*] and *RMS*[*z*_*r*_] with *γ* = 1 as

22 $${\gamma }_{ini}=\frac{RMS[z(x,y;1)]}{RMS\left\{{z}_{d}(x,y)-b(x,y)\ast { {\mathcal F} }^{-1}\left[\frac{{Z}_{d}(u,v)}{\bar{B}(u,v;1)}\right]\right\}}.$$

#### Two-level iterative scheme

As shown in Algorithm 4.2.2, the inner iterations guarantee the non-negativity of *t* with the least increase of the total dwell time, which is further reduced by minimizing the DG size in the outer iterations.

**Algorithm 1:**
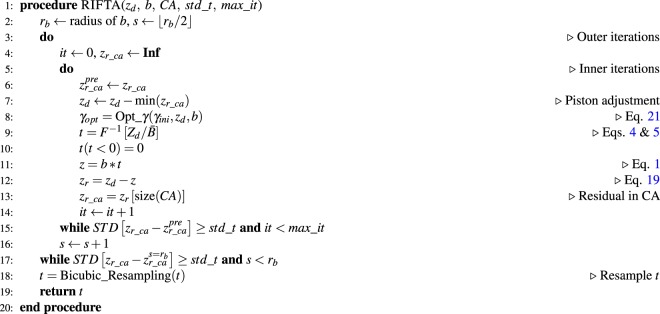
RIFTA dwell time algorithm.

##### Inner iterations 

If the lowest entry of *z*_*d*_ is outside CA, to ensure the non-negativity of the dwell time solution *t*, a piston should be added to adjust the entries in DG. This operation dominates the increase of total dwell time, since the piston may be over-added inadvertently. Therefore, in RIFTA, as shown by Lines 6 to 15 in Algorithm 4.2.2, the inner iterations only depend on the residuals in CA, ${z}_{r\_ca}$, to add pistons to DG. In each iteration, *z*_*d*_ is adjusted by a piston of ${\rm{\min }}({z}_{r\_ca})$, where min(·) represents the minimum entry in “·”. The negative entries in the calculated *t* in Line 9 are set as zeros in Line 10. As a result, it is always guaranteed that *z*_*d*_ is adjusted by the smallest (*i.e*. optimal) piston during the iterative calculations. The inner iterations are performed until the STD of the difference between the current and the previous residual maps in CA, *i.e*. $STD[{z}_{r\_ca}-{z}_{r\_ca}^{pre}]$, is less than the threshold *std_t* or the maximum number of iteration *max_it* is reached.

##### Outer iterations 

In the classical IBF algorithms^11–14^, DG is larger than CA with a size *s* on each side equal to the radius of *b*, *i.e. s* = *r*_*b*_. In this configuration, we can obtain the minimal residual in CA ${z}_{r\_ca}^{s={r}_{b}}$ from the inner iterations. However, we found that a much smaller *s* is sufficient to obtain a residual ${z}_{r\_ca}$ equivalent to ${z}_{r\_ca}^{s={r}_{b}}$ while the total dwell time is reduced. As shown in Algorithm 4.2.2, starting from $\lfloor {r}_{b}/2\rfloor$, the outer iterations keep searching the smallest *s* until the STD of ${z}_{r\_ca}-{z}_{r\_ca}^{s={r}_{b}}$ is less than the threshold *std_t*. In this study, *std_t* = 0.02 nm and *max_it* = 10 are used.

#### Bicubic resampling

Now we obtain the optimized dwell time *t*. However, it shares the same sampling interval with the measurement data *z*_*d*_, which is inconvenient for the practical IBF process. To add the flexibility of having different sampling intervals between metrology and fabrication, which has been realized in the matrix-based methods^10,11,13,17^, we use bicubic resampling to downsample *t* to flexible sampling intervals that the IBF process requires.

## References

[1] Yamauchi K (2011). Single-nanometer focusing of hard x-rays by kirkpatrick–baez mirrors. J. Physics: Condens. Matter.

[2] Yamauchi, K. *et al*. Focusing mirror for coherent hard x-rays. In *Synchrotron Light Sources and Free-Electron Lasers: Accelerator Physics, Instrumentation and Science Applications* (Springer International Publishing, 2016).

[3] Cheng, H. *Independent variables for optical surfacing systems* (Springer, 2016).

[4] Imanaka O, Okutomi M (1979). New concepts on surface finishing and its application to ceramicsrecent progress in ultra-fine finishing in japan. NBS Publ..

[5] Rückriem, R., Zeuner, M. & Köhler, R. P2. 2-fabrication of high-sensitivity pyroelectric sensors by ion beam etching. *Tagungsband* 551–554 (2016).

[6] Demmler M (2010). Ion beam figuring (ibf) for high precision optics. Adv. Fabrication Technol. Micro/Nano Opt. Photonics III.

[7] Idir M (2015). A one-dimensional ion beam figuring system for x-ray mirror fabrication. Rev. Sci. Instrum..

[8] Zhou L (2016). One-dimensional ion-beam figuring for grazing-incidence reflective optics. J. Synchrotron Radiat..

[9] Zhou L (2016). New figuring model based on surface slope profile for grazing-incidence reflective optics. J. Synchrotron Radiat..

[10] Wang T (2019). Study on an effective one-dimensional ion-beam figuring method. Opt. Express.

[11] Carnal CL, Egert CM, Hylton KW (1992). Advanced matrix-based algorithm for ion-beam milling of optical components. Curr. Dev. Optical Des. Optical Eng. II.

[12] Jiao C, Li S, Xie X (2009). Algorithm for ion beam figuring of low-gradient mirrors. Appl. Opt..

[13] Wu JF, Lu ZW, Zhang HX, Wang TS (2009). Dwell time algorithm in ion beam figuring. Appl. Opt..

[14] Wilson S, McNeil J (1987). Neutral ion beam figuring of large optical surfaces. Curr. Dev. Optical Eng. II.

[15] Drueding TW, Bifano TG, Fawcett SC (1995). Contouring algorithm for ion figuring. Precis. Eng..

[16] Schulze, C., Nestler, M. & Zeuner, M. Ion-beam figuring of x-ray mirrors. In Hull, T. B., Kim, D. W. & Hallibert, P. (eds.) *Astronomical Optics: Design, Manufacture, and Test of Space and Ground Systems II*, **11116**, 314–324, 10.1117/12.2530212. International Society for Optics and Photonics (SPIE, 2019).

[17] Zhou L, Dai Y, Xie X, Jiao C, Li S (2007). Model and method to determine dwell time in ion beam figuring. Nami Jishu yu Jingmi Gongcheng/Nanotechnology Precis. Eng..

[18] Lucy LB (1974). An iterative technique for the rectification of observed distributions. astronomical J..

[19] Nelder JA, Mead R (1965). A simplex method for function minimization. computer J..

[20] Mahajan VN (2010). Orthonormal aberration polynomials for anamorphic optical imaging systems with rectangular pupils. Appl. Opt..

[21] Tianyi, W. IBFest: Dwell time algorithm for ion beam figuring, 10.5281/zenodo.3715508 (2020).

[22] Richard G. Baraniuk. Inverse Filtering. http://www.owlnet.rice.edu/elec539/Projects99/BACH/proj2/inverse.html (1995).

[23] Lagarias JC, Reeds JA, Wright MH, Wright PE (1998). Convergence properties of the nelder–mead simplex method in low dimensions. SIAM J. Optim..

[24] Press, W. H., Teukolsky, S. A., Vetterling, W. T. & Flannery, B. P. *Numerical recipes 3rd edition: The art of scientific computing* (Cambridge university press, 2007).

